# SIGVerse: A Cloud-Based VR Platform for Research on Multimodal Human-Robot Interaction

**DOI:** 10.3389/frobt.2021.549360

**Published:** 2021-05-31

**Authors:** Tetsunari Inamura, Yoshiaki Mizuchi

**Affiliations:** ^1^ National Institute of Informatics, Tokyo, Japan; ^2^ Department of Informatics, The Graduate University for Advanced Studies (SOKENDAI), Tokyo, Japan

**Keywords:** virtual reality, software platform, social and embodied intelligence, dataset of interaction, robot competition

## Abstract

Research on Human-Robot Interaction (HRI) requires the substantial consideration of an experimental design, as well as a significant amount of time to practice the subject experiment. Recent technology in virtual reality (VR) can potentially address these time and effort challenges. The significant advantages of VR systems for HRI are: 1) cost reduction, as experimental facilities are not required in a real environment; 2) provision of the same environmental and embodied interaction conditions to test subjects; 3) visualization of arbitrary information and situations that cannot occur in reality, such as playback of past experiences, and 4) ease of access to an immersive and natural interface for robot/avatar teleoperations. Although VR tools with their features have been applied and developed in previous HRI research, all-encompassing tools or frameworks remain unavailable. In particular, the benefits of integration with cloud computing have not been comprehensively considered. Hence, the purpose of this study is to propose a research platform that can comprehensively provide the elements required for HRI research by integrating VR and cloud technologies. To realize a flexible and reusable system, we developed a real-time bridging mechanism between the robot operating system (ROS) and Unity. To confirm the feasibility of the system in a practical HRI scenario, we applied the proposed system to three case studies, including a robot competition named RoboCup@Home. *via* these case studies, we validated the system’s usefulness and its potential for the development and evaluation of social intelligence *via* multimodal HRI.

## 1 Introduction

Human-robot interaction (HRI) is one of the most active research interest in robotics and intelligent systems. Owing to the complexity of the HRI system, there are several challenges facing its research activities. One of such challenges is the collection of a dataset for machine learning in HRI (Amershi et al., 2014), which is required to learn and model human activities. A conventional strategy for assessing human activity in their interaction with robots is *via* video surveillance systems, such as motion capture systems. For example, Kanda (Kanda et al., 2010) developed a massive sensor network system to observe human activity in a shopping mall environment, over a period of 25 days. Another application of the interaction between a robot and children in an elementary school required approximately two months to collect the interaction dataset (Kanda et al., 2007). The significant cost of such an observation is a limitation of HRI research.

Consequently, experimental investigations involving virtual reality (VR) and simulations are garnering significant attention as potential methodologies for reducing the cost of data collection. Accordingly, various researchers have proposed the application of VR and simulation systems in the context of HRI. In addition to the desirable reduction in the cost of data collection that can be achieved, other significant advantages of this approach are: 1) cost reduction, as experimental facilities are not required in a real environment. 2) provision of similar environmental and embodied interaction conditions to test subjects (participants). 3) visualization of arbitrary information and situations that cannot occur in reality, such as playback of past experiences, and 4) easy access to an immersive and natural interface for robot/avatar teleoperation systems.

Although some systems and tools realize each of these functions, no system or framework can comprehensively consider all of them. In particular, crowdsourcing is a robust tool in recent HRI research, which is realized by linking VR systems with cloud computation; however, no platform can efficiently realize this function. The lack of such a system limits the promotion of HRI research. Accordingly, a middleware-like platform for HRI + VR systems, which is equivalent to the robot operating system (ROS) middleware, is required for the intelligent robot community. In this study, we propose SIGVerse, a system that can comprehensively realize all of the already mentioned functions.

**FIGURE 1:**
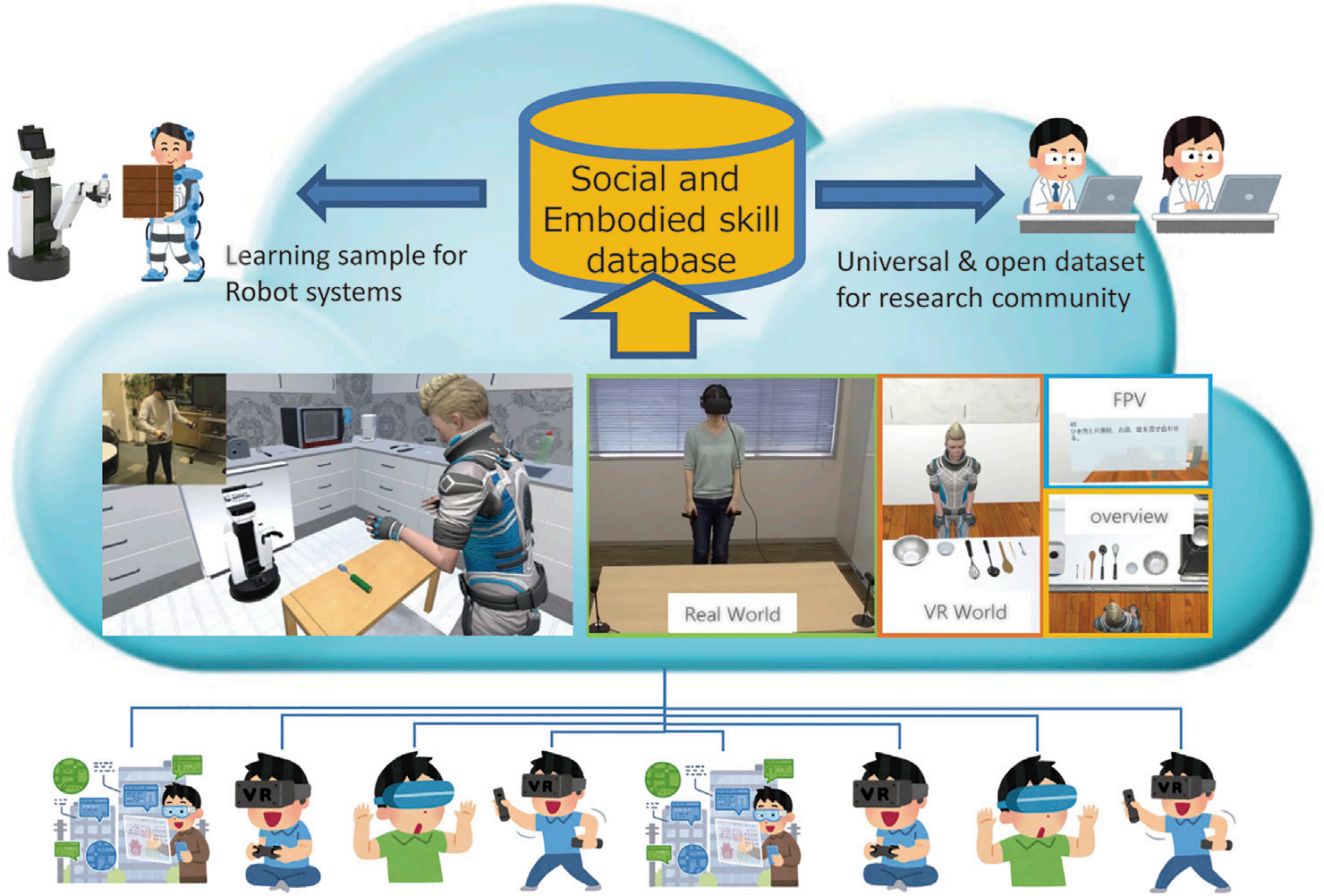
SIGVerse system concept. Arbitrary client users can participate in the HRI experiment *via* the Internet using VR devices.

The proposed system allows participants to participate in HRI experiments, which are set up in the VR environment *via* the Internet, without actually inviting them to the real laboratory or experimental field, as illustrated in Figure 1. Because the participants can log in to an avatar with the VR device and interact face-to-face with the virtual robot from the first-person’s view, most HRI experiments conducted so far can be realized in a VR environment. The time burden on the participants can also be distributed *via* crowdsourcing, based on the invitation of more participants.

The contributions of this study are as follows: 1) The proposed SIGVerse, which is an open software platform with high reusability, addresses four significant challenges: cost reduction of experimental environment construction, provision of the same experimental conditions to participants, reproduction of past experiences, and development of a base system for the natural interface of robot/avatar teleoperations. 2) It validates the effectiveness of these functions *via* case study implementation and verification. 3) It establishes a foundation for an objective and fair evaluation of HRI, primarily by deploying the proposed system in actual robot competitions.

In Chapter 2, we discuss related works from various perspectives on the advantages of VR in HRI. In Chapter 3, we comprehensively describe the system configuration and implementation method for realizing these functions. In Chapter 4, we present case studies using the significant functions, and in Chapter 5, we discuss the effectiveness of the proposed SIGVerse system, including the challenges that are difficult to address. Finally, Chapter 6 summarizes the findings and contributions of this study.

## 2 Related Works

### 2.1 Cost Reduction of Experimental Environment

Various physical functions of intelligent robots, such as learning strategies for grasping objects (Levine et al., 2018), manipulating flexible objects (e.g. cloth) (Yang et al., 2017), and automatic driving (Grigorescu et al., 2019), have been significantly improved by the recent development of machine learning technology. The common principle of these robot learning techniques is that they prepare a sample of body movements and decisions in the form of datasets. Some methods, such as Yang’s system (Yang et al., 2017), require only a few tens of data. However, the more complex the problem, the larger the amount of data required, and the required data in such cases are in the range of thousands to tens of thousands (Osentoski et al., 2010). This quantity of data is impractical to obtain in real environments. Although we can achieve autonomous learning by creating an environment in which robots can repeat object manipulation tasks, the situation differs if the learning target changes to interaction behavior with humans.

VR is also beneficial in collecting data on rare or dangerous situations. It has been adopted for training in disaster and accident evacuation scenarios (Sharma and Otunba, 2012) and driving simulators (Wiederhold and Wiederhold, 2010), which are too dangerous to reproduce in real environments. It does not only aid trainings and simulations, but also enables the data collation and analysis of human interaction behavior with robots in dangerous and rare situations (Robinette et al., 2019).

Based on these datasets, various multimodal behavioral datasets have been proposed for HRI datasets. The KIT motion dataset (Plappert et al., 2016) provides a combination of the modalities for motion pattern and natural language description. Movie datasets (Regneri et al., 2013; Sigurdsson et al., 2016) of human activities in daily life environments have been proposed. Regarding conversations, the collection of utterances and evaluation of communication in navigation tasks have been introduced (MacMahon et al., 2006; Vries et al., 2018; Mei et al., 2016). Owing to the research on natural language processing, the range of modality combinations is effective in the evaluation of behavior recognition and expansion in collaboration; however, it is limited. Because it also requires dedicated experimental environments and equipment to introduce more data, developing the open dataset in the research community is challenging.

### 2.2 Provision of a Uniform Experimental Condition for Participants

In cognitive psychological HRI research, the physical and social conditions of the participants in an experiment are strongly required to be uniform across participants and designed to be statistically analyzable (Coolican, 2017; Hoffman and Zhao, 2021). It is also important to solely control the experimental conditions to be compared, instead of other factors. However, it is challenging to completely unify such experimental conditions in a real environment. As a prerequisite for the design of experiments in real environments, substantial efforts are required to optimally unify lighting conditions and acoustic conditions, as well as the human behavior, other than the other participants in the experiment.

This unification challenge and control of conditions can be addressed using VR, which will be beneficial in ensuring the quality of the cognitive psychological research, and also in determining a clear baseline for evaluating the performance of interactive social robots.

For example, in the RoboCup@Home competition (Iocchi et al., 2015), which evaluates the quality of human-robot interaction, it is difficult to address this limitation. In the real competition field, it is difficult to completely unify the physical environmental conditions, such as the presence or absence of environmental noise during voice recognition and changes in lighting conditions during image recognition. The social and embodied behaviors of the participants participating in the experiment also varies from trial to trial (Scholtz et al., 2004). Therefore, evaluating quality with statistical reliability is a challenge.

Because human perception differs in many aspects between actual reality and VR, we cannot simply replace the experimental environment with VR (Wijnen et al., 2020). Therefore, it is necessary to consider several conditions, such as differences in distance perception (Li et al., 2019), gaze control (Duchowski et al., 2014; Sidenmark and Gellersen, 2019), and visual perception owing to a narrow field of view (Willemsen et al., 2009; Mizuchi and Inamura, 2018).

### 2.3 Multimodal user Interface for HRI in VR

Fang et al. developed a simple wearable motion measurement device (Fang et al., 2019) that can be worn on the human arm and hand, thereby enabling robot teleoperation with detailed accuracy. However, head-mounted displays (HMDs) have not been adopted to provide images from the robots’ perspective. In addition, because it is not a general-purpose consumer device, the participation of participants in a cloud environment is limited.

Lemaignan et al. proposed the application of SPARK, which enables social robots to assess the human-robot interaction condition and plan an appropriate behavioral response based on a spatiotemporal representation using three-dimensional (3D) geometric reconstructions from robot sensors (Lemaignan et al., 2017). They also expanded the system to share the representation among a group of service robots, such as UNDERWORLDS (Lemaignan et al., 2018). Because the main contribution of both systems is to establish an internal representation of the world for social robots, the systems do not support a user interface that shares this representation with humans. Simulation of the social interaction behavior between robot systems and real humans is an important function of service robots; however, these systems require actual robots for the assessment and planning of social interactions. Hence, real-time human participation in a virtual HRI scenario is the objective of our study.

Another related work is the digital twin (El Saddik, 2018), which creates a digital replica of the real world, including humans, robot agents, and environments. An application of the digital twin in HRI is the investigation and optimization of the interaction/interface system and the design of robot systems The manufacturing engineering field has recently focused on this technology; consequently, several software systems have been proposed (Bilberg and Malik, 2019; Kuts et al., 2019). However, the main focus of these attempts is the real-time reproduction of the physical world. In addition, the real-time participation of humans in the virtual world has not been thoroughly discussed and developed.

In conventional VR systems, focus has often been placed on the user interface to render the audiovisual and tactile information perceived by humans more realistic. In the case of application of VR systems in robot teleoperation, the presence of a human being in the VR space is not necessary because users control the robots directly. However, in the case of HRI in VR, it is important to note that users log in to the system and assume an avatar (virtual human) that interacts in real time with the virtual robot with spatial and embodied cognition. It is also critical to ensure that the virtual robot can observe the avatar to understand its behavior.

### 2.4 Base for Teleoperation Systems

In recent years, VR systems (Lipton et al., 2018; Whitney et al., 2020) and skill learning based on teleoperation demonstrations (Mandlekar et al., 2018; Zhang et al., 2018) have been adopted to actively perform robot teleoperations with the aim of cost reduction.

VR and simulations are often used to compensate for the lack of information when operating robots in extreme environments such as space robotics (Yoon et al., 2004) and nuclear power plant maintenance (Pruks et al., 2018), as delays and communication breakdowns are expected to occur. In this case, it is necessary to predict and visualize the future *via* simulations (Clarke et al., 2007). Therefore, it is necessary to cooperate with prediction systems that differ from the VR user interfaces and visualization subsystems.

In cognitive psychological HRI research, the Wizard of Oz (WoZ) method is often adopted to operate a robot (Komatsubara et al., 2018; Riek, 2012). In this case, the goal is to control the robot’s behavior, such that it appears natural to the participants, rather than controlling the robot for motor learning remotely. To achieve this, the operator must control the robot by grasping the images observed from the robot’s viewpoint and the participants’ behavior in real time. In addition, the robot must be able to interact with the participants without delays, as the robot may interact with the participants by voice or physical means, depending on the situation.

The problem common to these applications is the challenge in grasping the situation from the robot’s viewpoint, depending on the type of task. Therefore, it would be beneficial to apply VR to reproduce images from other viewpoints (Okura et al., 2014), such as images from a camera installed on the ceiling or images from an omnidirectional camera.

### 2.5 System Versatility and Scalability

Bazzano *et al.* developed a VR system for service robots (Bazzano et al., 2016). This system provides a virtual office environment and an immersive VR interface to test the interaction between virtual robots and real humans. Because the software of a service robot should be developed in the C# script on the Unity system, the compatibility among real robots is low.

From the perspective of platforms, several open source platforms for artificial intelligence (AI) agents in indoor environments, such as Malmo (Johnson et al., 2016), MINOS (Savva et al., 2017), AI2THOR (Kolve et al., 2017), have been proposed. These projects provide free and open software platforms that enable general users to participate in the research and development of intelligent agent systems, in which the difficulty in building robot agents and 3D environment models is eliminated. Because the concept of the systems involves the easy development of an autonomous agent system, the control of AI agents is limited to sending simple commands by script, without using an ROS. Furthermore, the systems do not support real-time interaction between real users and AI agents because the primary target is the interaction between AI agents and the environment.

In this study, we focus on the realization of an open platform to collect and leverage multimodal-interaction-experience data that were collected in daily life environments and require embodied social interaction.

## 3 SIGVerse: A Cloud-Based VR Platform

In this section, we introduce the concept of a could-based VR system to accelerate research on HRI.

For example, collaborative cooking tasks and dialogue-management systems dealing with vague utterances and gestures, as well as gazing behaviors are assumed to be target situations that involve HRI scenarios. In these situations, a robot must observe and learn the social behaviors of the humans with which it interacts and solves ambiguities based on past interaction experiences. In these complex environments, the robot collects the following multimodal data:

1. Physical motion/gestures during interaction (including gaze information)
2. Visual information (i.e., image viewed by the agents)
3. Spatial information (i.e., positions of agents and objects)
4. Voice interaction (i.e., utterance of agents)

Furthermore, the following functions must be provided:

i). Users are able to login to avatars in the VR environment from anywhere
ii). Multiple users can simultaneously login to the same VR scene *via* the Internet
iii). Time-series multimodal interaction data can be recorded and replayed
iv). Control programs of real robots can be attached to virtual robots

Functions (i) to (iii) are based on the real-time participation of humans in the virtual environment, which is yet to be discussed in conventional robot simulators. Function (iv) is required for the efficient development of robot software, which can be used in both real and virtual environments. Therefore, the support of robotic middleware is essential. Function (iii) requires high quality graphics function and computational power for the physical simulation. The reusability of the ready-made 3D robot model and the daily life environment is also essential in constructing a variety of virtual environments for HRI experiments. Because we have several types of data formats for the robot model, compatibility is another important function that ensures efficient development. Table 1 presents the performance of existing related systems from the perspective of these required functions.

**TABLE 1:** Functions and limitations of related systems.

Platform	Graphics	Physics/dynamics	3D model format	Ready-made model/environment	Robotic middleware	Immersion of human
Gazebo (Koenig and Howard (2004))	OGRE	ODE, bullet, simbody, DART	SDF/URDF, STL, OBJ, collada	40 + robot models, 7 competitions	ROS	Not supported
USARSim (Lewis et al. (2007))	Unreal engine 2, unreal engine 3, unreal development kit		ut2, ut3, udk	5 + robot modes, RoboCup rescue, RoboCup soccer	Player, ROS	Not supported
V-REP (Rohmer et al. (2013))	OpenGL	Bullet,ODE, vortex, Newton	OBJ, STL, DXF, 3DS, collada, URDF	30 + robot models	ROS	Not supported
Choreonoid (Nakaoka (2012))	OpenGL	AIST engine, ODE, bullet, PhysX	Body, VRML	A few robot models	OpenRTM	Not supported
Open-HRP (Kanehiro et al. (2004))	Java3D	ODE, bullet	VRML	A few robot models	OpenRTM	Not supported
Webots (Michel (2004))	WREN (OpenGL)	ODE	WBT, VRML, X3D	50 + robot models, 500 + objects, 6 environments	ROS, NaoQI	Not supported
OpenRAVE (Diankov and Kuffner (2008))	Coin3D, OpenSceneGraph	ODE, bullet	XML, VRML, OBJ, collada	10 + robot models	ROS, YARP	Not supported
MINOS (Savva et al. (2017))	WebGL	N/A	Unknown	SUNCG, Matterport3D	N/A	Not supported
Project Malmo (Johnson et al. (2016))	Minecraft		Unknown	MARLÖ competition	N/A	Not supported
AI2THOR (Kolve et al. (2017))	Unity		FBX, collada, 3DS, DXF, OBJ, ...	200 + environments, 2,600 + objects	N/A	Not supported
VirtualHome (Puig et al. (2018))	Unity		FBX, collada, 3DS, DXF, OBJ, ...	6 environments, 350 + object models, knowledge base	N/A	Not supported
DeepMind lab (Beattie et al. (2016))	Quake III arena		Unknown	Several games	N/A	Not supported
OpenAI gym (Brockman et al. (2016))	MuJoCo, atari, Box2D, 15 + simulation environments		Unknown^a^	Unknown^a^	N/A^b^	Not supported
iCub-HRI (Fischer et al. (2018))	N/A		N/A	1 robot model	YARP	Not supported
SIGVerse (Ver.2) (Inamura (2010))	OGRE	ODE	X3D, VRML	A few robot model	N/A	Supported
SIGVerse (Ver.3)	Unity		FBX, collada, 3DS, DXF, OBJ...	5 + robot models, 200 + objects, 40 + environments	ROS	Supported

a Depends on the adopted simulation environment.

b ROS is solely supported in a third-party environment gym-gazebo.

Our previous system (SIGVerse ver.2) (Inamura, 2010) has been applied in studies such as the analysis of human behavior (Ramirez-Amaro et al., 2014), learning of spatial concepts (Taniguchi et al., 2016), and VR-based rehabilitation (Inamura et al., 2017). These studies employed multimodal data (1)–(4) and functions (i)–(iii); however, the reusability of the conventional SIGVerse is restricted because it does not adopt ROS as its application programming interface (API). Therefore, we updated the system to maintain functions (i)–(iii) and realize function (iv). The following subsections present the software architecture that realizes these functions.

### 3.1 Architecture of the SIGVerse

**FIGURE 2:**
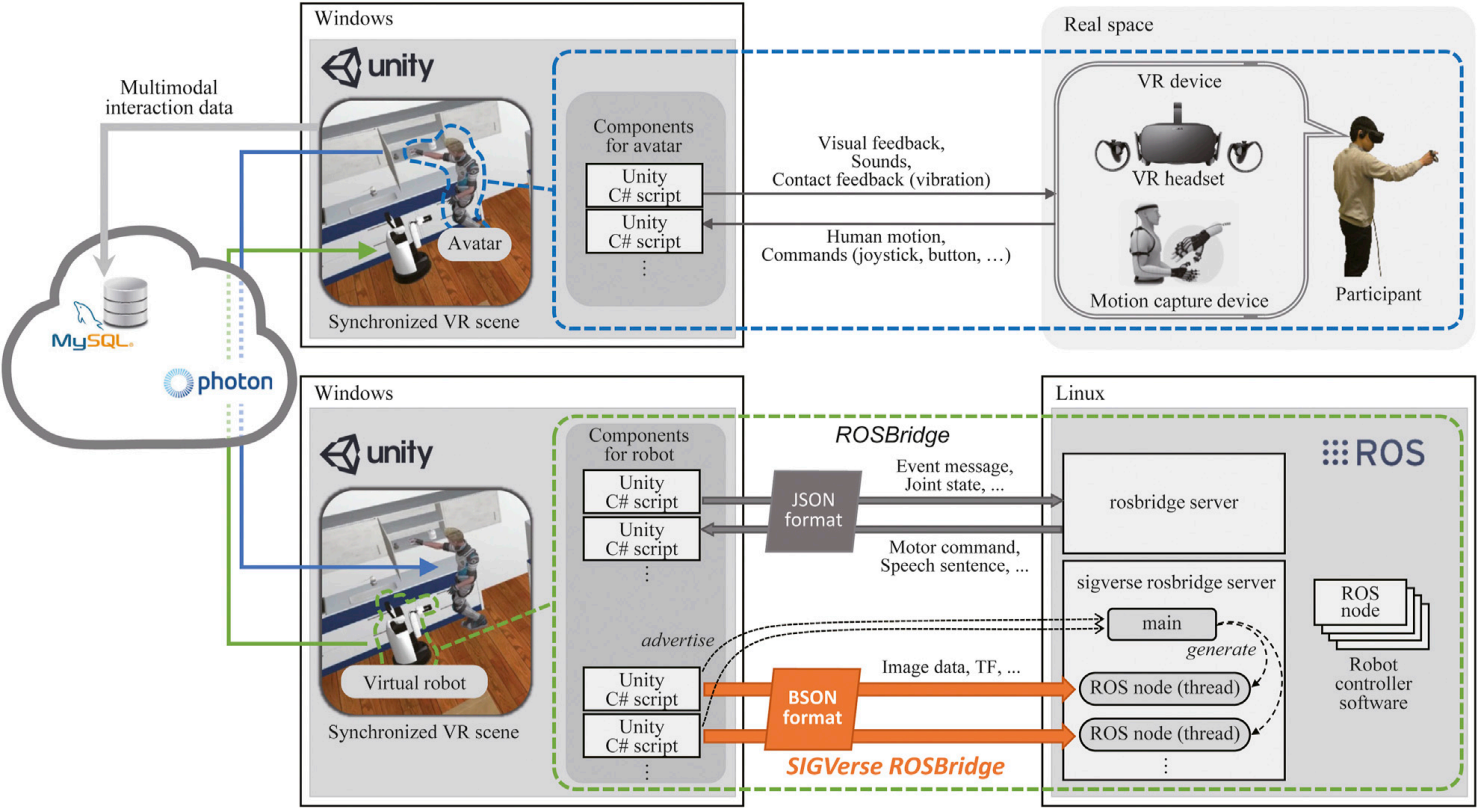
Software architecture of the SIGVerse system.

The detailed architecture of SIGVerse (Version. 3), which includes a participant and a robot, is illustrated in Figure 2. SIGVerse is a server/client system that is based on a third-party networking technology (i.e., Photon Realtime). The server and clients share the same scene, which is composed of 3D object models such as avatars, robots, and furniture. By transferring the information of registered objects *via* the Internet, the events in each scene can be synchronized.

The participant can login to an avatar *via* VR devices, such as HMDs, hand-tracking controllers, audio devices, and motion capture devices. According to the input from these VR devices, the behavior of the participant is reflected on the avatar by Unity scripts. Perceptual information, such as perspective visual feedback, is provided to the participant. Therefore, the participant can interact with the virtual environment in a manner similar to a real environment.

The proposed VR simulation system has a bridging mechanism between the ROS and Unity. Software for virtual robot control can be reused in real robots without modification, and vice versa.

The information required for reproducing multimodal interaction experiences is stored on a cloud server as a large-scale dataset of embodied and social information. By sharing this information, users can reproduce and analyze multimodal interactions after the experiment.

### 3.2 Mechanism for Connecting ROS and Unity

To control a robot in a VR environment, sensory feedback and robot commands should be transferred between Unity scripts and ROS nodes. The most important factor in realizing the integration of ROS and unity is the communication protocol between them. Software systems for bridging ROS and Unity have been proposed by Hu et al. (Hu and Meng, 2016) and Downey et al. (Codd-Downey et al., 2014). For these systems, the motor commands and sensor information are transferred using a rosbridge. However, if users attempt to transfer a massive amount of sensor information from Unity, such as camera images, transfer speed is inhibited. Previous works (Codd-Downey et al., 2014; Hu and Meng, 2016) did not consider how to transfer camera images in real time; accordingly, a novel technique for realizing real-time transfer based on the binary JavaScript object notation (BSON) format, using a TCP/IP connection, is proposed in the following section.

As a ROS functionality, the rosbridge framework provides a JavaScript object notation (JSON) API and a WebSocket server to ensure communication between an ROS program and an external non-ROS program. JSON is a text-based data exchange format that represents pairs of keywords and values. Although the rosbridge protocol ensures sending and receiving ROS messages, its performance in transferring large JSON data, such as images, is insufficient and cannot satisfy real-time sensor feedback. Accordingly, a specific server (sigverse_rosbridge_server) is implemented to communicate large data volumes. To speed up communication, the BSON format was employed instead of JSON. BSON is a binary-encoded serialization with a JSON-like format. The use of BSON offers the following advantages: reduction in communication data size to less than that of text-based data, independence of the conversion process between text and binary, and representation of data as key-value pairs that are compatible with ROS messages. When ROS messages are advertised by Unity scripts, the main thread of the sigverse_rosbridge_server generates a new thread for each topic as an ROS node. Each thread receives ROS messages from the Unity scripts and publishes them in the ROS nodes of the robot controller as ROS topic messages.

Siemens and the ROS community have proposed the software module, ROS#, to support communication between ROS and Unity^1^. ROS# employs the rosbridge to send and receive control commands, including the status of the robot. Recently, rosbridge has provided optional functionality for transferring data using the BSON format. However, the data exchange format, as well as the efficient and fast emulation of sensor data, is important for real-time transfer of sensor data. As another unique feature, SIGVerse can generate the uncompressed binary data of image data (i.e., raw data with the exact memory structure as actual camera devices installed on real robots) at high speeds using Unity’s functionalities. Detailed descriptions of the sensor emulation process are provided in the Supplementary Appendix.

**FIGURE 3:**
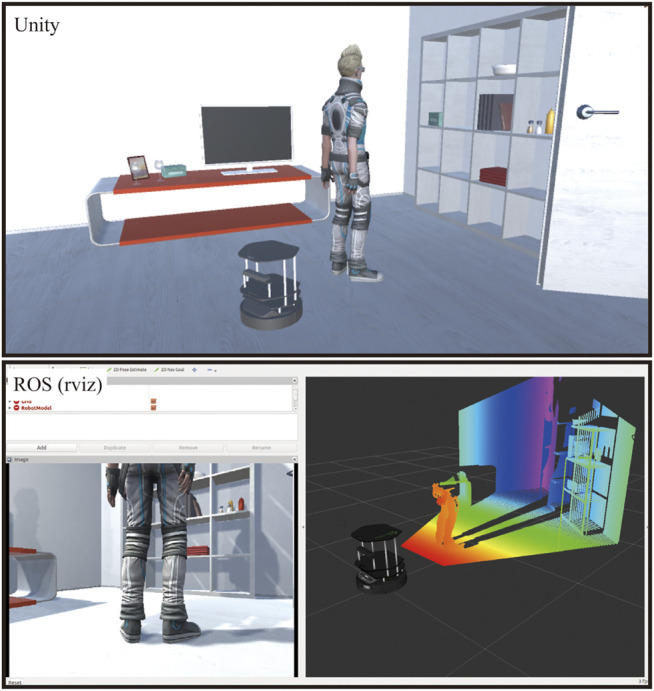
Virtual RGB image and depth data transferred from Unity to ROS. The top image illustrates a VR scene, which includes a robot with an RGB-D sensor, while the bottom figure illustrates an RGB image and depth data received in the ROS.

We evaluated the data transfer performance (Mizuchi and Inamura, 2017) to compare the proposed method with the conventional JSON-based method. The experimental conditions presented in Figure 3 were adopted for the investigation, where a mobile robot tracked a walking person using an RGB-D sensor. A PC with a Xeon E5-2687W CPU and GeForce GTX TITAN X GPU were used in this evaluation. The size of the raw RGB-D frame was 1.5 MB. The average frequencies of RGB-D data are presented in Table 2. The JSON communication was insufficient in satisfying the real-time requirement for HRI, even when a high-end computer was employed.

**TABLE 2:** Frequencies of virtual RGB-D data depending on protocols.

WebSocket with JSON	TCP/IP with BSON
(Conventional rosbridge)	(sigverse_rosbridge)
0.55 [fps]	57.60 [fps]

### 3.3 User Interface for HRI in VR

The Oculus Rift is available as the default VR device when a human logs into an avatar. The user wears the HMD on the head and controls the avatar in VR by grasping the hand controllers, called Oculus Touch, with both hands. The position and posture of the HMD and hand controllers are measured in real time, and reflected in the avatar’s head and hands. Because there is a difference in body structure between the user and the avatar, only the avatar’s head and end-effector are visualized. If an application requires the visualization of the posture of the entire body, the joint angles of the upper body are calculated *via* inverse kinematics based on the position and orientation of the Oculus Touch sensors, or a Kinect sensor used in combination with the Oculus Touch sensors.

**FIGURE 4:**
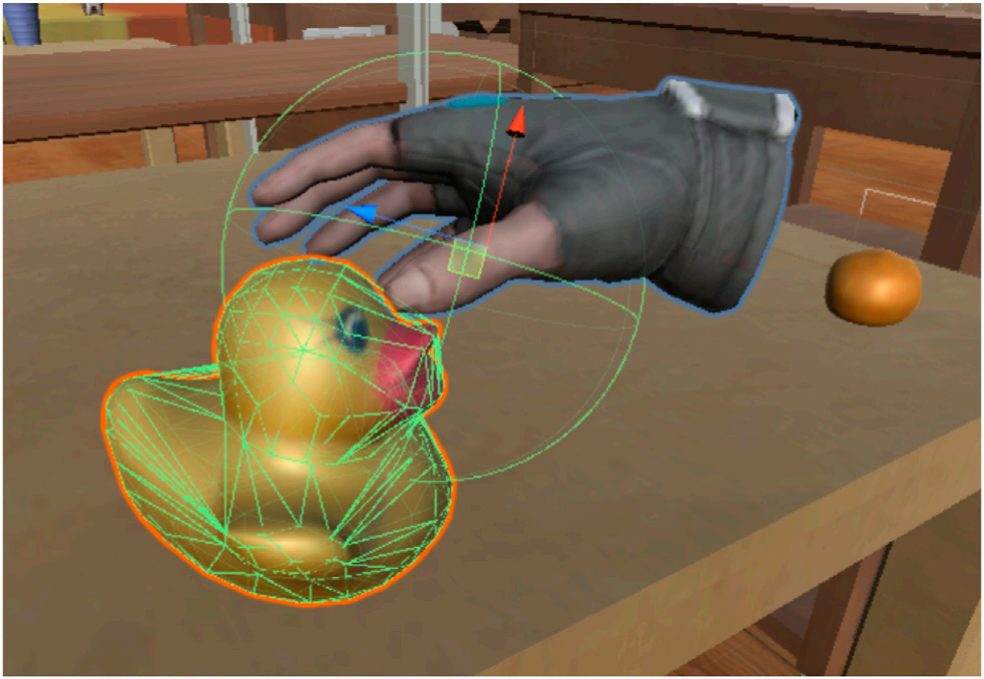
Mesh Collider configuration to realize simple object grasping function by human avatar.

To grasp objects in VR, we adopted a Unity asset called NewtonVR^2^. The script NVR Interactable Item was attached to the graspable object, and the script *NVR Hand* was attached to the human avatar’s hand. We also attached a mesh structure for collision detection, called a Mesh Collider, as illustrated in Figure 4. A mesh structure based on the shape of the object was attached to each object. A simple sphere mesh was then attached to the hands of the avatar with a radius of 7 cm. When the user pushed the trigger button, the system investigated the overlap of these meshes. If the meshes overlap, the grasp is considered successful, and the object is connected to the end-effector. Although the Newton VR function controls the object’s grasping status, it is not affected by the physics simulation, such as gravity.

To realize object manipulation with constraint conditions, such as drawers and hinge joint doors in VR, we attached Configurable Joint and Hinge Joint asset scripts to the object and set the constraint conditions. By setting the range of motion with the linear limit option for the configurable joint and the limit option for the hinge joint, we achieved natural object manipulation. Attaching the NVR Interactable Item script to the door handle allowed the object to be grasped in the same way as general objects.

The time delay was approximately 40–60 ms, which is required to render the 3D images of the robot/avatar/environment states in the VR space, and project the image onto the HMD. Because this delay time may trigger VR sickness, it is necessary to consider reducing the computational load when an HRI experiment is performed. Major factors that increase the computational load are the type and amount of data to be recorded. The data recording settings described in Section 3.5 need to be varied according to the robot task and application objective.

### 3.4 Configuration of the Cloud-Based VR Space for HRI

To enable general users to participate in an HRI experiment from an arbitrary location, we developed a cloud-based VR function on SIGVerse. Each user logs into an avatar *via* a client computer, which has a Unity process for VR devices. To control the behavior of the target robot, the user’s computer connects to another computer, which has another Unity process. The internal states of all VR scenes are synchronized *via* the Internet based on Photon Realtime, which is a universal software module for integrating different game engines. For the simple use case, each computer (Unity process) was connected to a cloud server provided by Photon Inc.

Figure 2 also illustrates the cloud configuration employed in SIGVerse. One computer (Unity process) was assigned to each user/robot to realize the complex interaction between multiple robots and users.

**FIGURE 5:**
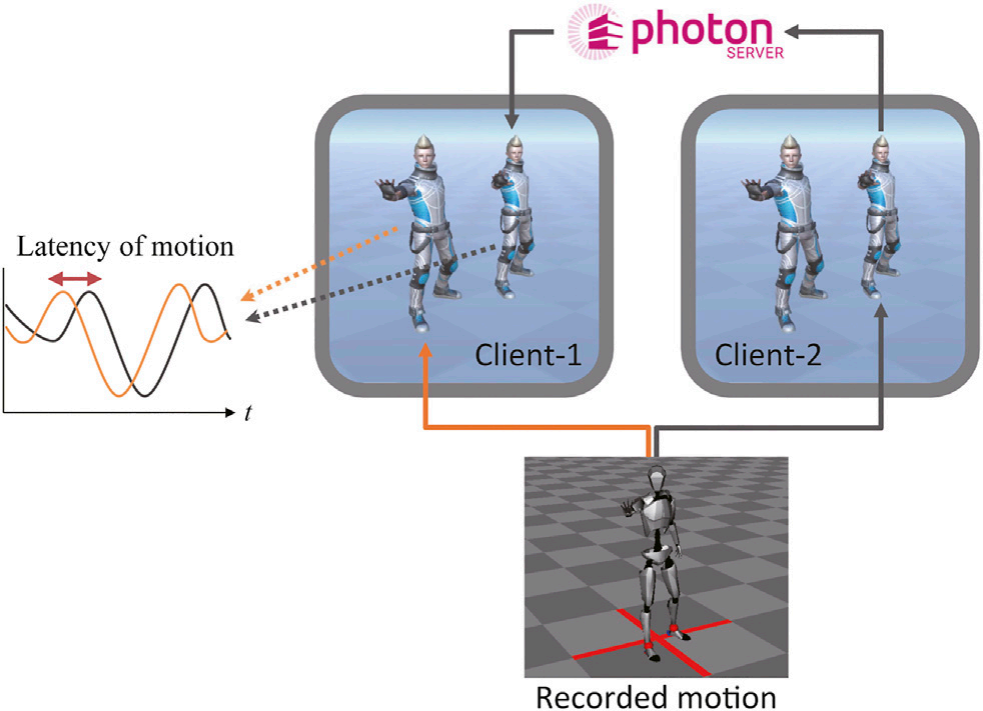
Configuration of latency evaluation.

We measured the latency between a local avatar and other avatars, whose motions were synchronized using a local Photon server. The configuration of the latency evaluation is presented in Figure 5. The broadcasted motion player software (Axis Neuron) pre-recorded motion data *via* a TCP/IP connection. Because all the clients were located in the same place, the motion difference between local avatars was negligible. The postures of the non-local avatars were synchronized with those of the corresponding avatars in each client. The position and rotation of 56 joints, including the fingers, were synchronized for each avatar. Latency was evaluated in 2-client, 4-client, and 8-client cases.

**FIGURE 6:**
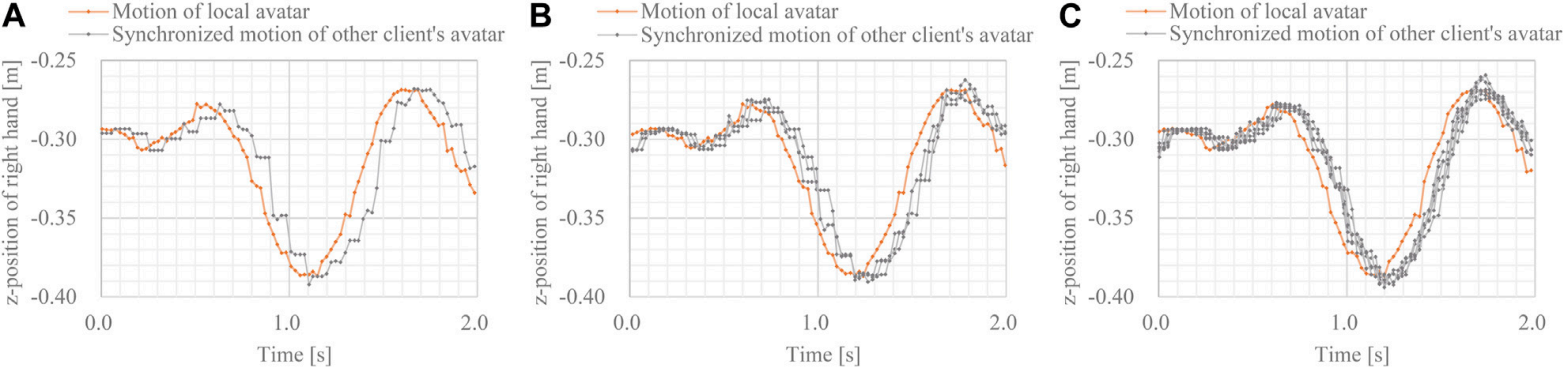
Movements of local and synchronized avatars **(A)** movements in 2-client case **(B)** movements in 4-client case, and **(C)** movements in 8-client case.

The *z*-position of the right-hand side in each case is shown in Figure 6. Although the motions of the non-local avatars were slightly disturbed and delayed, their motions were synchronized. Regardless of the number of clients, the latency of synchronized motions was approximately 70 ms. This latency is sufficiently low to allow multimodal interaction and cooperative tasks among multiple robots and avatars in a cloud-based VR space.

### 3.5 Database Subsystem for Recording Human-Robot Interaction

The SIGVerse system has a database subsystem that records multimodal HRI behavior, and the targets of the recording consist of physical and cognitive interactions. As the physical interaction, the system records the actions of the robot and avatar (time-series data of joint angles and positions of the robot and avatar), and time-series data of the status of objects that are manipulated by the robot and the avatar. As the cognitive interaction, the system records verbal conversations between the robot and avatar. Because the recorded data can be stored in the MySQL server, a client can reproduce the physical and cognitive interactions in the VR environment from anywhere *via* the Internet.

Although the sensor signals measured by the robot are useful information as HRI records, the database subsystem does not record sensor signals because the computational load for simulating and recording the sensors is high. For example, HSR has five cameras of four types: an RGBD camera, a camera attached to the end-effector, a wide-angle camera on the head, and a stereo camera. If all of these camera images were recorded at the specified frame rate, the amount of data would be approximately 500 Mbps. Because it is impractical to record all of the camera data, we implemented a playback mode. The playback mode provides a function to reproduce the sensor signals from the recorded behavior of the robot, avatar, and objects, instead of recording the sensor signals directly. For example, in the case of learning by demonstration, when a robot learns a behavior by observing a human body movement and referring to the camera image obtained at that time, only the user’s body movement was recorded. After observing and recording the body movement, the robot used the playback mode to emulate the camera image and referred to the relationship between the camera image and body motions during learning. Therefore, SIGVerse is both a VR interface for humans and an experience reproduction simulator for robots.

## 4 Case Studies

### 4.1 Robot Competition

A better and more effective method for evaluating the performance of HRI is *via* robot competitions, such as RoboCup@Home (Iocchi et al., 2015). Because the organization of a robot competition requires a considerable amount of time and human resources, simulation technologies are often employed to reduce costs. Although RoboCup Soccer and RoboCup Rescue have simulation leagues, RoboCup@Home does not have a simulation league. One of the reasons for realizing the RoboCup@Home simulation is the need for interactions between real and virtual robots. This problem can be solved using SIGVerse. Although the competition participants do not need to learn about the VR system, they should concentrate on software development on the ROS framework. The product of the developed robot software can be easily applied to a real robot system.

Because it is difficult to change the rulebook and competition design of RoboCup, we organized a VR-based HRI competition in the World Robot Summit, Service Category, Partner Robot Challenge^3^, which was held in Tokyo, in October 2018 (Okada et al., 2019). The following subsections present two representative tasks in the competition based on the proposed system.

#### 4.1.1 Task 1: Interactive Cleanup

**FIGURE 7:**
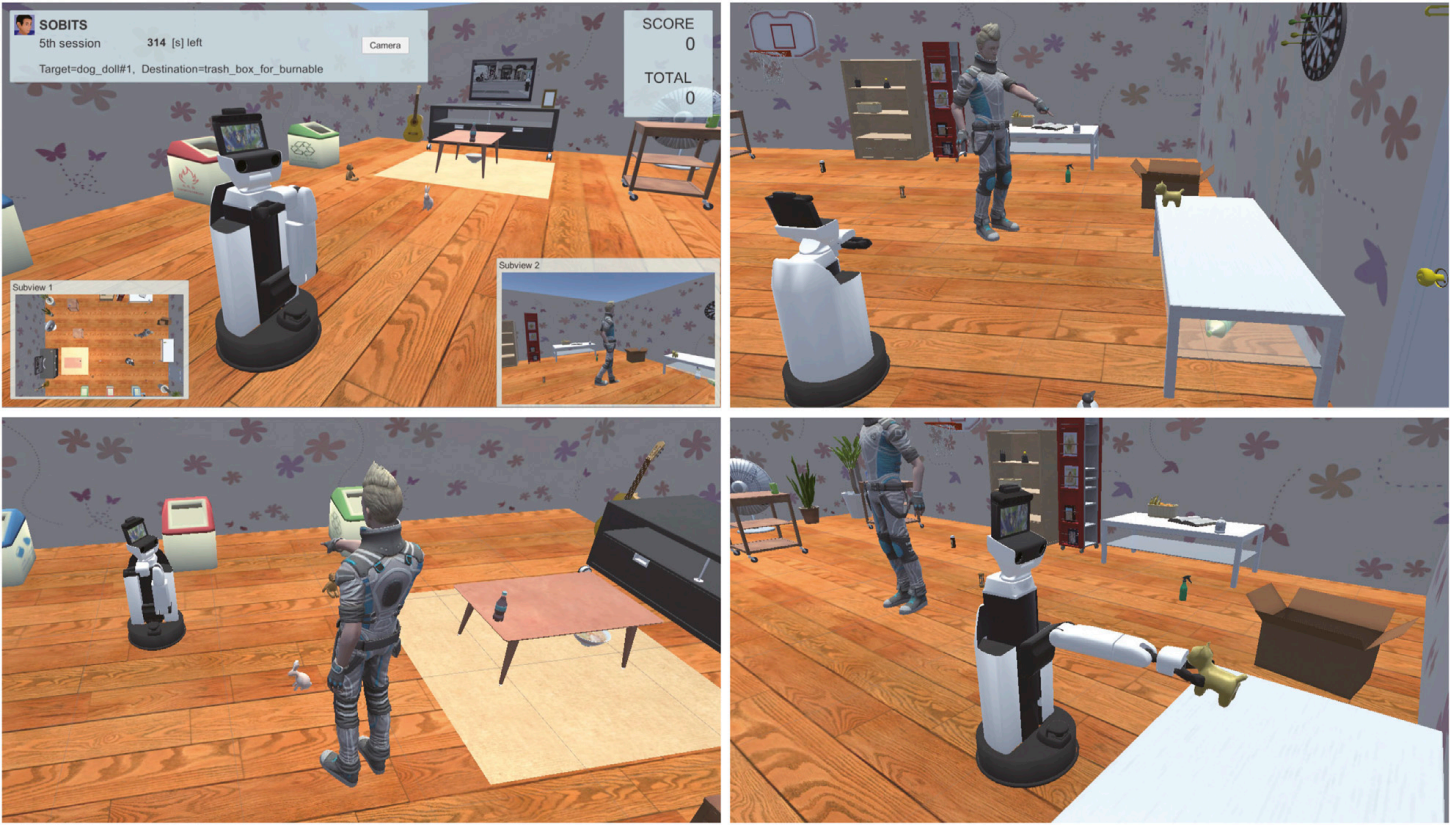
Screenshot of the *Interactive CleanUp* task.

Figure 7 In this task, the robot is required to clean up the room as instructed by a human using pointing gestures. The human points to a target object anywhere in the room and then gestures to a trash can, a cupboard, or any other location to be cleaned up. Because the competition field is a large room, humans have to walk around and make pointing gestures. Therefore, it is not enough for the robot to recognize the gestures’ video, but the robot also needs to track the moving human and observe gestures.

Gesture recognition is a basic function for understanding human behavior. The image dataset (Wan et al., 2016) and competition (Escalera et al., 2013) have played an important role in this area over the past several decades. The gesture recognition functions required for intelligent robots that work with people include both label recognition, and it also follows a moving human and observation of the gesture from an easy-to-view perspective. Furthermore, the pointing target object is recognized from the spatial relationship between the object that exists in the environment and the human.

It was impossible to provide precisely the same conditions to multiple competing teams when we performed the competition in an actual field. Although similar competition tasks existed in RoboCup@Home, only one robot could interact with a human at a time. Therefore, the human had to repeat similar pointing gestures severally. However, because it is impossible to precisely reproduce the same posture and movements, it is not easy to evaluate the performance with strict fairness. In addition, increasing the number of trials would cost too much time; hence, it was sometimes necessary to evaluate the performance with only one trial in the worst case.

#### 4.1.2 Task 2: Human Navigation

**FIGURE 8:**
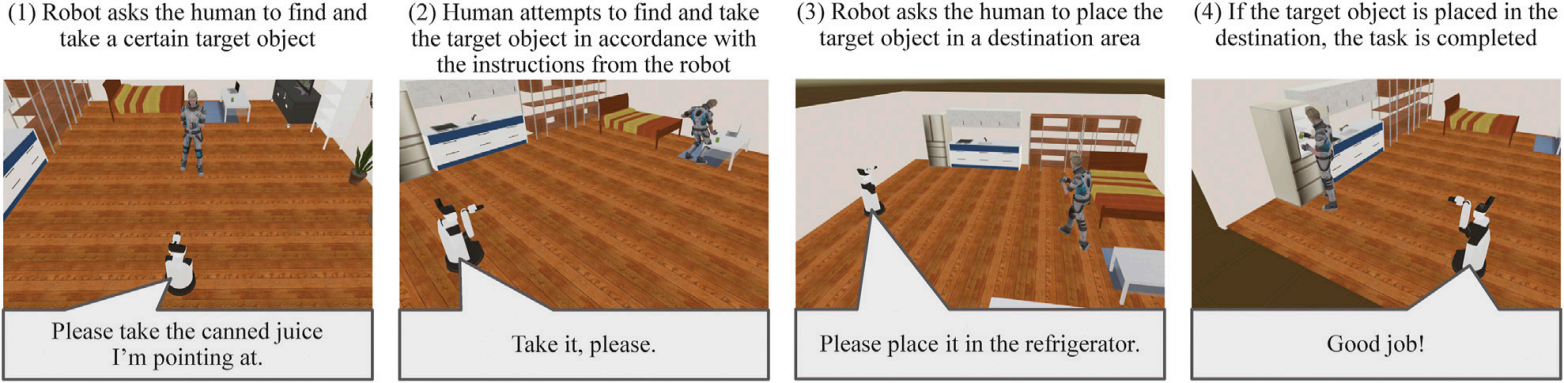
Sequence of events in the *Human Navigation* task.

**FIGURE 9:**
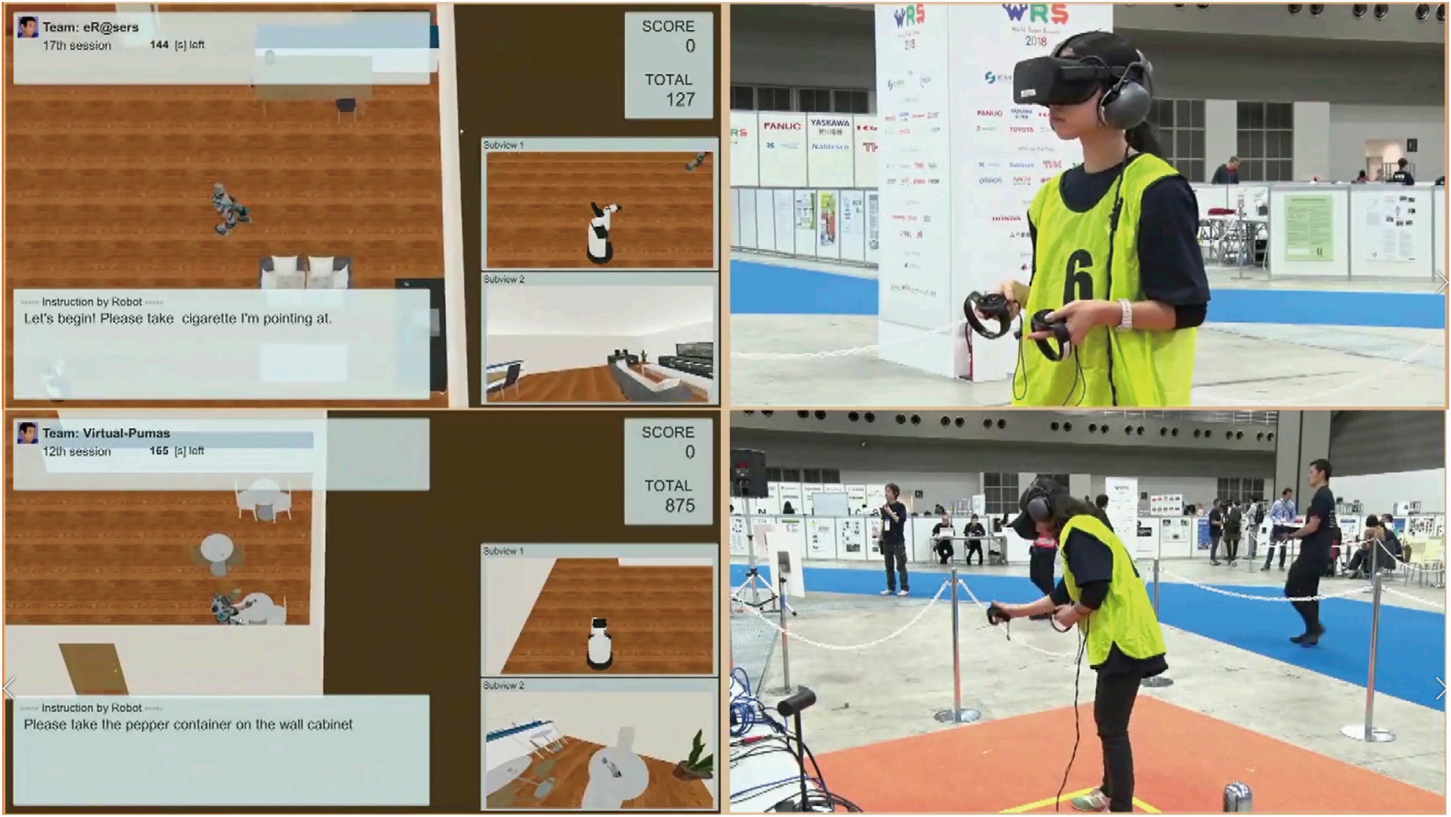
Screenshot of the *Human Navigation* task. Written informed consent was obtained from the test subject for the publication of any potentially identifiable images or data included in this article.

Here, we focus on a task named Human Navigation (Inamura and Mizuchi, 2017), in which the robot has to generate friendly and simple natural language expressions to guide people to perform several tasks in a daily environment, for the evaluation of HRI in the VR environment. The roles of the robot and the user are opposite to those in the conventional task, such as the roles of understanding and achieving a request given by users. The robot has to provide natural language instructions to inform a person to carry a certain target object to a certain destination, for instance, “Please take the cup on the table in front of you to the second drawer in the kitchen.” In this task, the human operator (test subject) logged into an avatar in a VR space and followed the instructions. The test subject then attempted to pick up the target object and take it to the destination using virtual reality devices by following the instructions from the robot, as illustrated in Figures 8, 9. The time required to complete this transportation was measured and applied to calculate the points. The team that generated the easiest and most natural instructions for a person to follow received more points.

According to the rulebook^4^ of this competition, we evaluated the reaction of the test subjects based on approach to addressing the utterance of the robot. A basic analysis of the interaction’s effectiveness was performed by the required time. If the instruction from the robot is unfriendly, the test subjects tend to be confused and exhaust a substantial amount of time in completing the task.

The significant features of SIGVerse that support this task are its abilities to record all human and robot actions in real time and switch between various environments. In this task, the intelligent robot’s performance is evaluated not only by the robot’s behavior, but also by the human’s reaction. The measurement and evaluation targets are the number of questions asked by the human, number of human failures, and time required for success. Although it is possible to perform the same task in a real environment, it would require attaching markers to the test subjects and introducing a motion capture system that covers the entire playing field, which is expensive. Otherwise, a human referee would have to constantly monitor the subject’s behavior to score the behavior. One of the advantages of VR is that the reactions can be measured and analyzed using a simple VR interface device.

**FIGURE 10:**
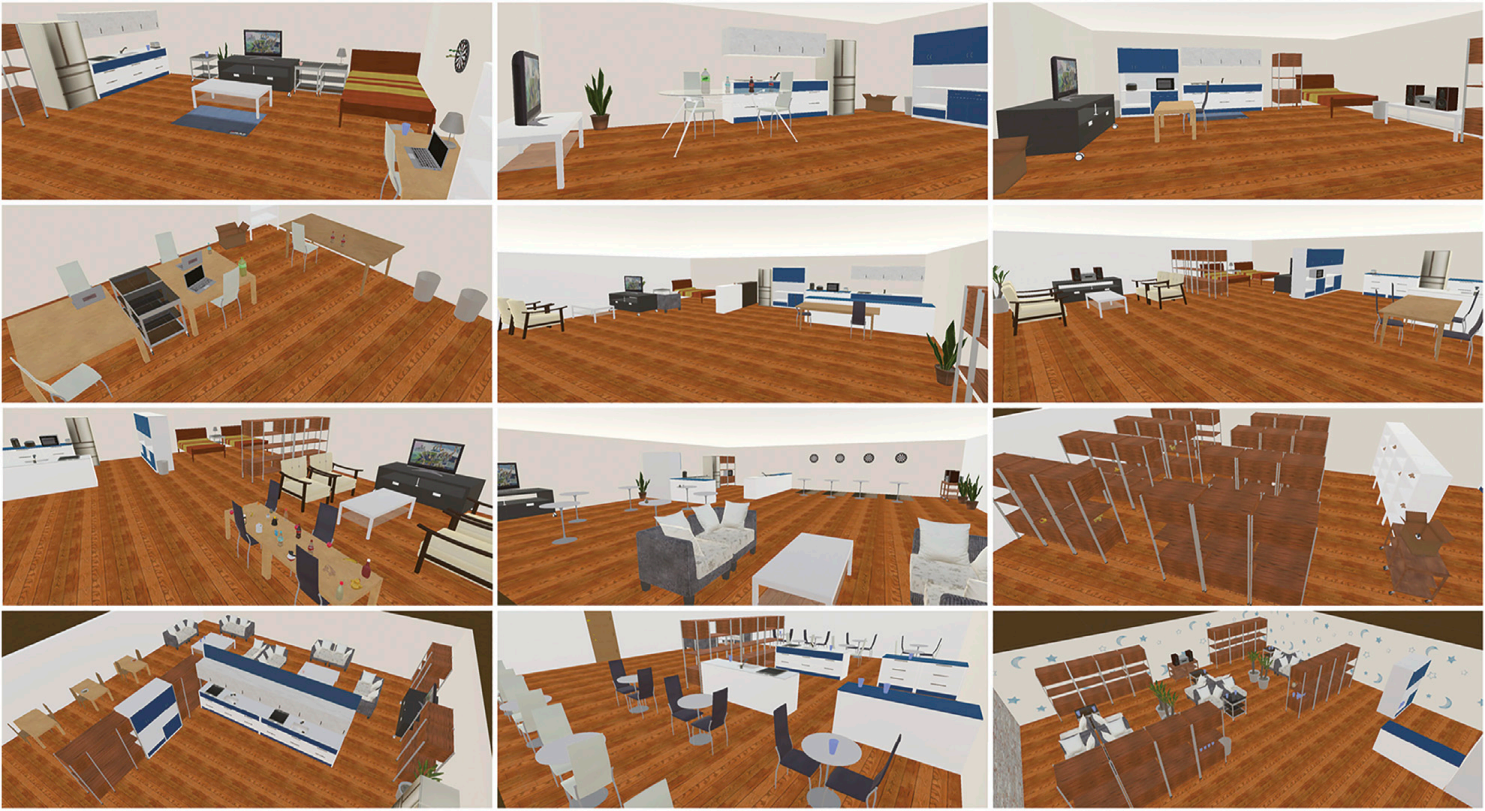
Part of the room layouts used in the *Human Navigation* task.

In addition, similar to the interactive cleanup task described above, the advantage of using SIGVerse is that we can statistically evaluate human behaviors in a wide variety of environments. When performing a similar task in a real environment, it is not easy to change the layout of the room owing to time limitations. Therefore, the subject remembers the layout of the environment as the number of trials increases. The ease of interpreting verbal explanations fluctuates between cases where the subject has prior knowledge of the environment and cases where the subject does not. In VR, however, the subject can be introduced into a completely unknown environment, whereby the learning effect can be eliminated and fair competition conditions can be ensured. We prepared 28 room layouts for the competition held in 2018; a part of the layout is illustrated in Figure 10.

#### 4.1.3 Competition on a Cloud System

The COVID-19 pandemic has made it difficult to conduct research on the HRI. Consequently, many robot competitions have been canceled in the past year. To address this challenge, we organized an online and virtual robot competition^5^ based on the SIGVerse system in 2020 (Inamura et al., 2021).

**FIGURE 11:**
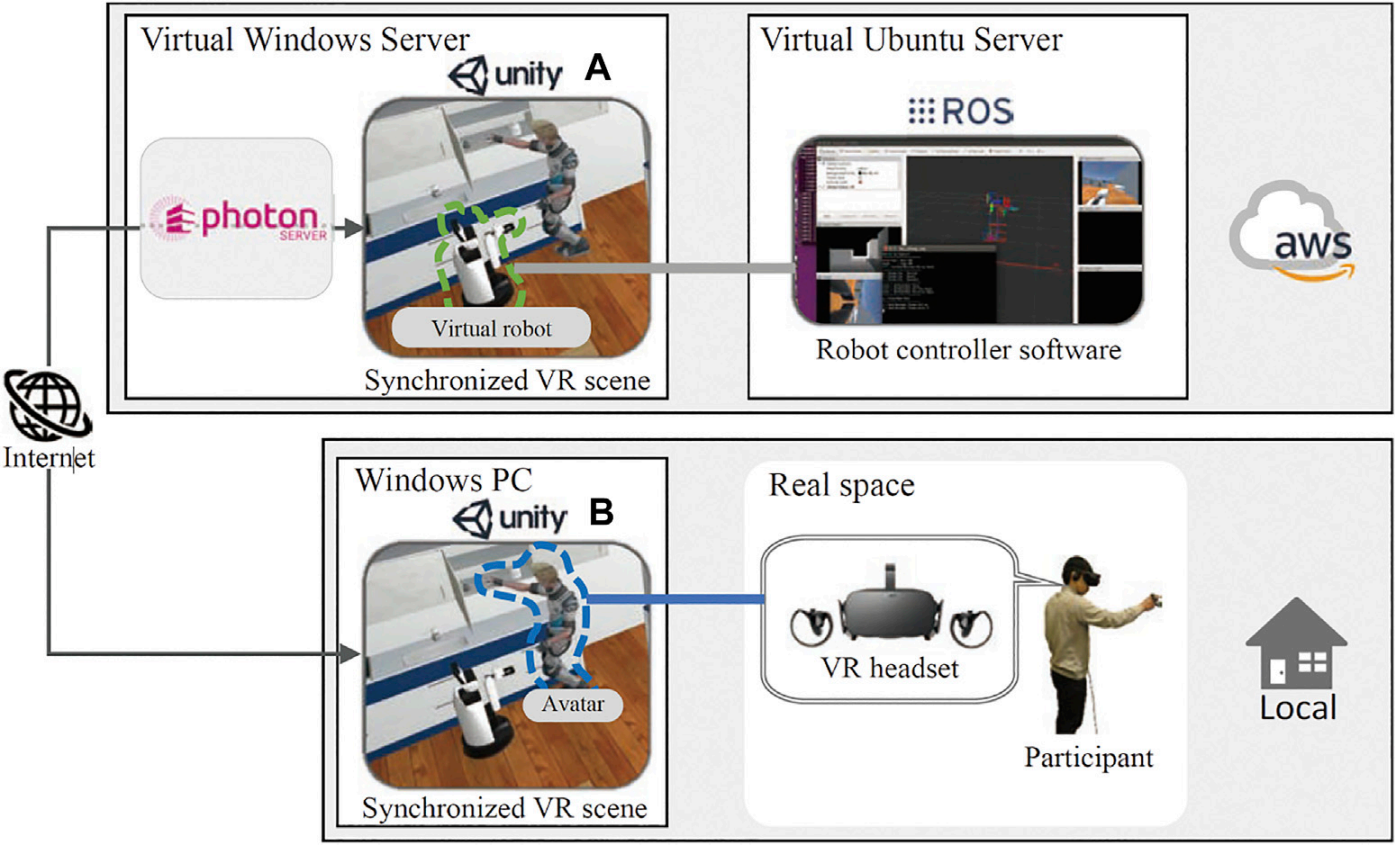
Overview of the cloud-based VR platform to perform HRI experiments.

In the conventional competition described in Sections 4.1.1, Sections 4.1.2, we brought the server to the competition venue and built a local network at the venue. In this study, because all participants and subjects needed to participate in the competition from home, we set up a SIGVerse competition field on the Amazon Elastic Compute Cloud (Amazon EC2)^6^, which is a subsidiary of the Amazon Web Service (AWS)^7^. The competitor teams submitted the robot software to the AWS, and the human navigation participants wore VR devices at home to participate in the experiment. The software configuration is illustrated in Figure 11.

The VR environment in which the robot’s control software was connected a) ran on the cloud server. Another VR environment in which the VR interface was provided for test subjects b) ran sin each user’s local environment. The events executed in each VR environment, such as the avatar’s body movements, robot movements, speech interaction, and object grasping, were synchronized *via* the Internet using Photon^8^, which enabled real-time physical interaction between the subject and the virtual robot. We imported the ready-made Photon Unity Networking (PUN) asset^9^ to the Unity project, which was used to build VR scenes. Although using Photon Cloud^10^ helps to connect VR scenes easily, we set up a photon server on the virtual Windows server to minimize delays in data communication.

Because it is difficult to establish the same experimental conditions in light of varying Internet connection quality at the subject’s residence, human navigation was conducted as an exhibition. Intearctive Cleanup and other competition tasks can be conducted online.

### 4.2 Modeling of Subjective Evaluation of HRI Quality

The interaction ability of mobile robots in the human navigation task was primarily evaluated by the time required to achieve the task. However, other factors were adopted as the evaluation target, such as the number of used words, frequency of the pointing gesture, and length of the trajectory of the avatar’s behavior. Designing an objective criteria for evaluation is difficult because a substantial number of factors are evaluated subjectively in a daily life environment. Although the best evaluation method involves asking several referees to score the performance in several trials, human navigation was evaluated by a certain regulation, such as a positive point for “desirable behavior” and a negative point for “unfriendly behavior,” which is described in the rulebook from a subjective viewpoint. We have addressed this challenge to determine the dominant factor for the evaluation of the interaction behavior in the HRI dataset (Mizuchi and Inamura, 2020).

The approach is to have a third party evaluate the quality of human-robot interaction, and model the relationship between the subjective evaluation points and the physical and social behaviors of humans and robots. Regarding the behavior of humans and robots, various factors and parameters need to be examined. For example, candidate targets for recording and analyzing include the direction of human/robot pointing gestures, information on objects in that pointing direction, physical movements during walking and object manipulation by human/robot, changes in object states owing to object manipulation, movement trajectories of human/robot, gaze direction of human/robot, information on objects beyond the gaze, and the number and frequency of speech. If we attempt to record all of these data in real time while the competition is in progress, the VR computer load will be too high, and it will be difficult to ensure real-time performance.

In addition, a situation may arise in which one wants to investigate a new parameter to be evaluated after the competition is over. In such a case, it would be necessary to repeat the competition if the data are not recorded, which is inefficient. Therefore, only the minimum necessary data, i.e., physical motions of humans and robots, including those of objects, should be recorded. As described in Chapter 3, we did not record the sensor signals of the robot, but reproduced the sensor signals in the playback mode by revisiting it if necessary. For example, the camera image data acquired by the robot at the moment of human speech were reproduced in the playback mode.

**FIGURE 12:**
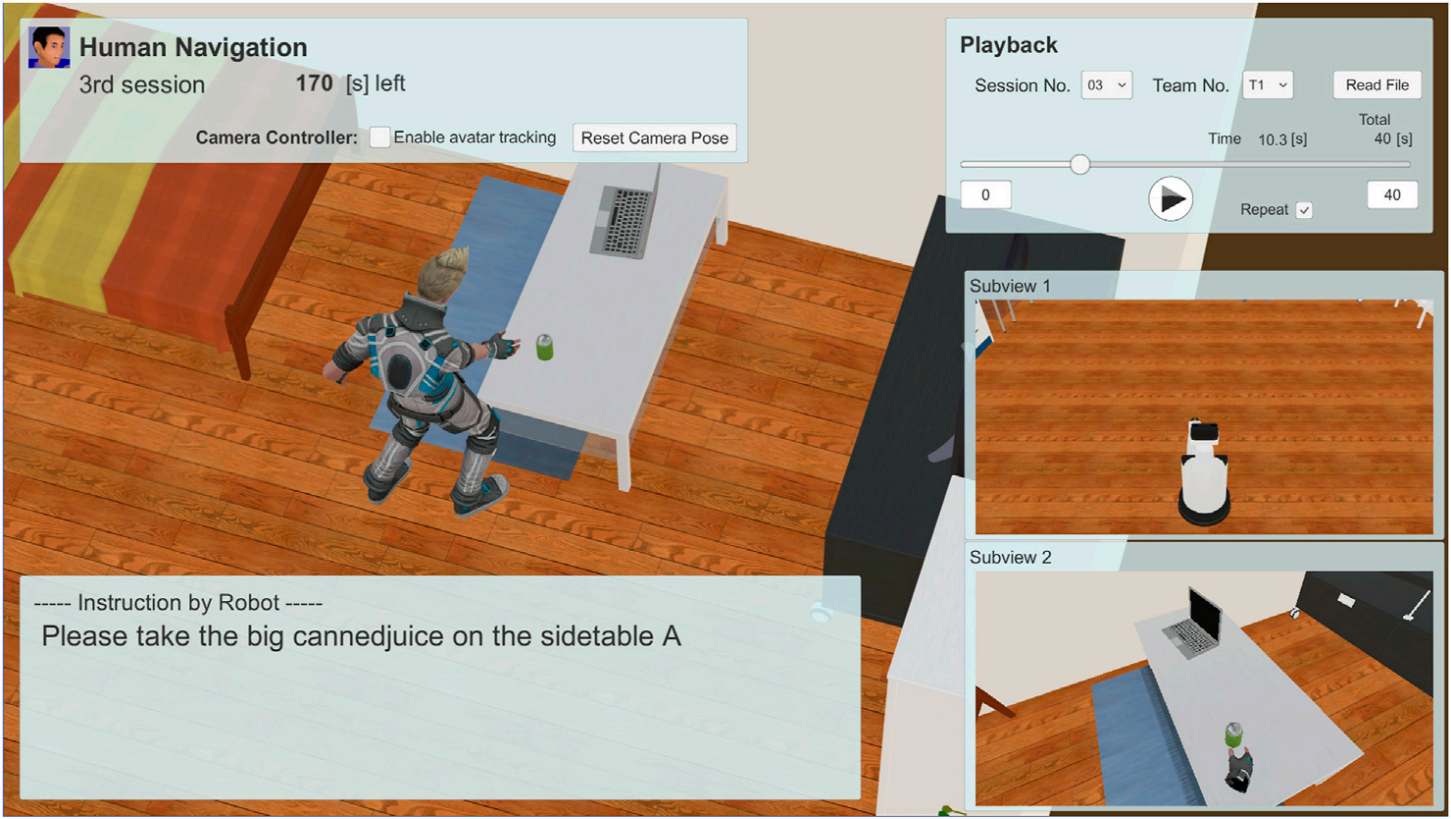
Screenshot of the playback system for subjective evaluation by third-parties.

After recording the human-robot interaction behavior, we asked the third person, who was not involved in the competition, to evaluate the quality of the interaction. Because the HRI behavior was played back in the VR system, the evaluators could check the detailed behavior by changing the viewpoint, rewinding the past behavior, and repeating the important scene, which is similar to the recent video assistant referee technology that is used in soccer games. This is another advantage of the playback mode both for robot sensor reproduction, and also for a flexible user interface in the subjective evaluation of HRI quality. Figure 12 shows a screenshot of the interface provided to the evaluator that graded the interaction quality. The “Playback” sub-window shown in the upper right area of Figure 12 represents the interface *via* which prior scenes could be chosen. The evaluator could also use this interface to adjust the viewpoint position and direction *via* mouse operation. This evaluation was conducted through crowdsourcing. Evaluators were asked to download software based on SIGVerse to observe the interaction log, which contained the recorded interactions between the participants and virtual robot. The observation could be carried out through a 2D screen, following which the evaluators had to enter their evaluation values. The only equipment that the evaluators had to arrange for was a standard Windows PC to download and execute the software. The evaluators were paid 1000 JPY per hour of work. The evaluator required approximately the same amount of time as the length of the history to observe the interaction log, and a few additional minutes to evaluate each session.

A 5-point Likert scale questionnaire was adopted for the evaluation. We use the following phrase for the questionnaire: “Interaction between the robot and test subject was efficient (Good: 5–Bad: 1).” We collected evaluation data from 196 sessions using 16 evaluators. To estimate the evaluation results as the objective variable, we selected 10 explanatory variables shown in Table 3. These parameters were designed after robot competition. Accordingly we developed a software to extract these parameters using the playback mode of SIGVerse. We also conducted multiple regression analysis to estimate the 5-point Likert scale evaluation using the following equation.

$$r=\alpha +\overset{n}{\underset{i=1}{\sum }}\beta_{i}\cdot x_{i},$$ (1)

where *r* is the grade of the questionnaire, *α* is the intercept value, $\beta_{i}$ is coefficient of *i*-th explainable variable, *i* is the index of the explainable variance, and $x_{i}$ is the measurement value of *i*-th explainable variable. The obtained coefficients for each explanatory variable are shown in Table 3.

**TABLE 3:** Regression coefficients obtained for each objective factors (Mizuchi and Inamura, 2020).

Explanatory variable	Notation of coefficient	Coefficient value
Time_task_comp: Time taken to complete the task	$\beta_{time\_task\_comp}$	−0.0145^a^
Time_grasp: Time taken to complete grasping of the target object	$\beta_{time\_grasp}$	−0.0022^a^
Num_incorr_grasp: Number of incorrect object-grasps	$\beta_{num\_incorr\_grasp}$	−0.0017
Any_inst: Whether the robot was able to generate any instructions	$\beta_{any\_inst}$	0.8518^a^
Num_inst: Number of instructions given by the robot	$\beta_{num\_inst}$	0.0207^b^
Num_word_sec: Number of words per second used in the instructions	$\beta_{num\_word\_sec}$	0.0266
Num_request: Number of instruction-requests given by the test subject	$\beta_{num\_request}$	−0.0026
Num_gesture: Number of pointing gestures given by the robot	$\beta_{num\_gesture}$	0.1071^b^
Nist_sec: Distance traveled by the test subject	$\beta_{dist\_sec}$	0.2882^d^
Face_dir_sec: Changes of face directions by the test subject	$\beta_{face\_dir\_sec}$	0.4470^d^
Adjusted	$R^{2}$	0.9500

Where significance codes.

a p < 0.001.

b p < 0.01.

c p < 0.05.

d p < 0.1.

The results obtained indicate that the subjective evaluation result was significantly influenced by the distance traveled by the test subjects and the changes in the gaze directions of the test subjects (Mizuchi and Inamura, 2020). The coefficient for the explanatory variable any_inst has a large value, which is a variable indicating whether or not the robot generated instructions, corresponding to the software bug that interrupted the robot behavior. Therefore, the two aforementioned factors are the significant variables. The importance of these two parameters was not anticipated before competition. Therefore, it was impossible to prepare a software to measure these data.

We compared the quality of the evaluation criterion between the results of the regression analysis and those of the rulebook that was used in the robot competition. The ranking determined by the evaluation criterion, which was calculated by the proposed method, differed from the ranking determined by the rulebook and was similar to the ranking determined by a third party. Because the parameters are easily measured in the VR environment, an automatic evaluation of the HRI behavior in the robot competition is easier without a subjective assessment by the referee. Owing to the high-cost of recording the behavior in the real robot competition, the proposed VR system would be one of the key technologies for evaluating the performance of a social robot in a real environment.

### 4.3 Motion Learning by Demonstration

Another advantage of the proposed system is the ease of behavior collection for robot learning. Motion learning by demonstration is one of the major applications in robot learning that requires human motion, as well as an interaction process between humans and the environment.

**FIGURE 13:**
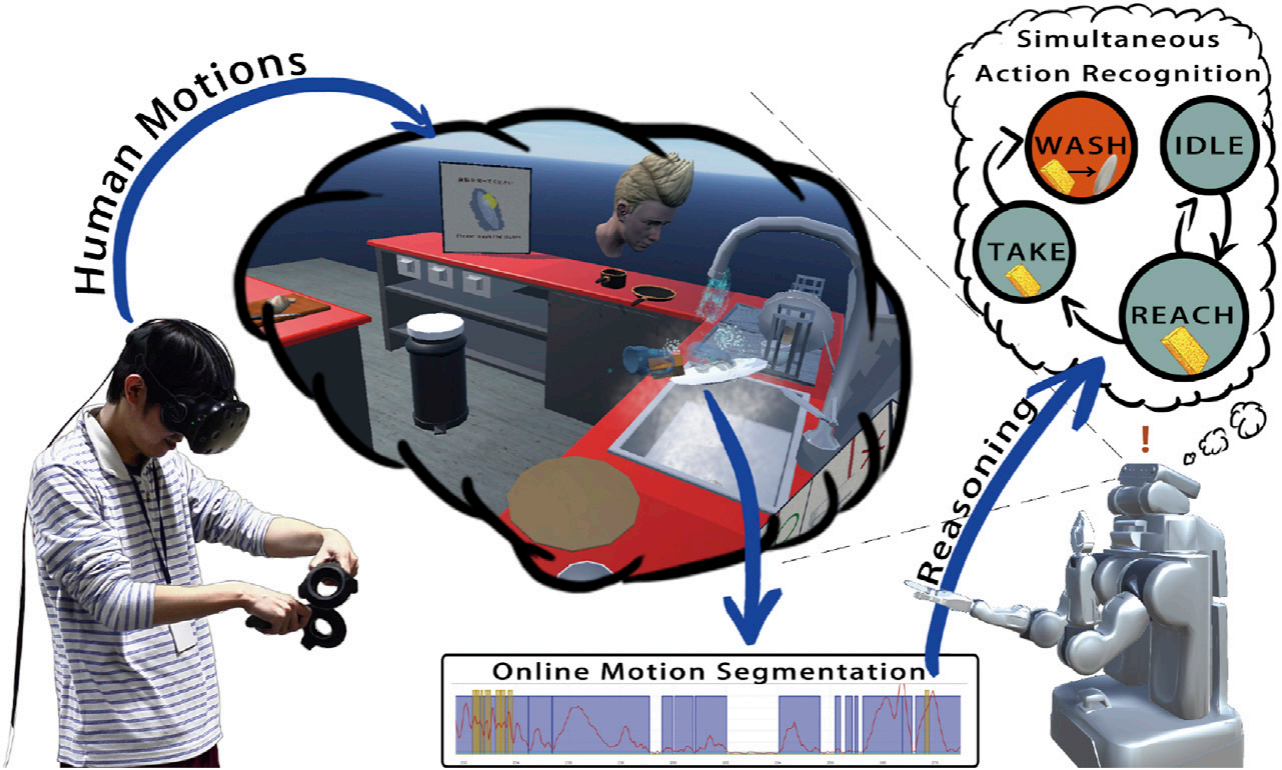
Behavior learning for washing dish task in a virtual environment (Bates et al., 2017).

Figure 13 Bates et al. proposed a virtual experimental environment for learning the dish washing behavior (Bates et al., 2017) based on the SIGVerse system. The participants logged into the VR environment and operated dishes and a sponge by hand devices, such as Oculus Touch. The motion patterns and state transition history of the related objects were recorded, and a semantic representation was learned from the collected data. Finally, a robot imitates the dish washing behavior in the real world with reference to the semantic representation, although the robot has never observed human behavior in the real world.

Vasco et al. proposed a motion learning method based on our system (Vasco et al., 2019). They attempted to make the robot system learn the motion concept, which includes motion patterns and also related information, such as tools and objects operated by the motion and location information where the motion is performed. Our system was used to collect motion data in various situations within a short time.

In both applications, the target of the learning is not only the motion pattern, but also the interaction process between the environments and objects. In addition, the focus is the effect of performance on the environment. The virtual environment provides an easy recording function for motion patterns and the state transition processes of virtual environments and objects. Although motion capture systems and object tracking functions are available in the real world, the cost of building a real field environment is still expensive for researchers.

## 5 Discussion

### 5.1 Implications from the Case Studies

#### 5.1.1 HRI Competition

The robotics competition conducted not only ensured an objective, fair, and statistically reliable HRI environment, but also indicated that cloud-based VR can potentially address the COVID-19 challenge through exhibitions. We evaluated cognitive aspects of human behavior. By introducing bio-signal measurement technology, it is possible to construct HRI datasets that include psychological evaluation and mental load. Because psychological analysis in this HRI research is supported almost entirely by experiments in real environments, the proposed system could have significant ripple effects.

#### 5.1.2 Modeling of Subjective Evaluation of HRI Quality

Sixteen evaluators participated in the subjective evaluation, which is not a large number; however, the evaluation was conducted by crowdsourcing. Because it is easy to scale up the number of evaluators *via* a cloud-based VR system, the proposed system is useful for subjective evaluation from a broader range of viewpoints. In addition, because the interaction quality is frequently evaluated in HRI research, the ability to perform such evaluations in VR can accelerate HRI research.

The discussion on domestic robots with applications in daily life has recently focused on the intelligence of service robots, such as the generating-instructions-in-virtual-environments (GIVE) challenge (Striegnitz et al., 2011) and visual questioning and answering (VQA) tasks (Das et al., 2018). These studies address the history of natural language interaction, the physical behavior of the robot/agent, and the 3D environment. However, they are limited by the restricted embodiment of the user’s avatar. In addition to the physical action of robots, the gestures and cognitive reactions of the human avatar to the robot’s behavior are significant factors in determining robot intelligence.

Whether the evaluation *via* the 2D interface is the same as that *via* the 3D immersive interface is an important and interesting research question. Because we could not distribute HMDs to all the evaluators as crowdworkers this time due to the time limitation, this will be explored as part of future work.

#### 5.1.3 Motion Learning by Demonstration

Demonstration by teleoperation is one of the most conventional ways of transferring human skills to robots. In fact, in the ROBOTURK system (Mandlekar et al., 2018), the robot teleoperation system is implemented by a simple mobile application, and human skills are collected and learned by a robot *via* crowdsourcing. In contrast, the advantages of SIGVerse are: 1) it allows the user to control any avatar based on the position and orientation of hand devices and HMDs, and 2) it allows the operator log in to both humanoid avatars and virtual robots. Therefore, SIGVerse can be used as a pre-learning tool for an actual robot to perform imitation learning by observing human behavior with a camera. In addition, the system can also be used to learn the behavior of another robot by observing the behavior of the operated robot (Ramirez-Amaro et al., 2014). This versatility is another advantage of the SIGVerse system.

### 5.2 Limitation

Because a physics simulation is performed in the Unity system, its complex simulations, such as friction force, manipulation of soft materials, and fluid dynamics, are limitations. Additionally, a standard 3D shape model of the robot, such as the URDF utilized in Gazebo, is not easily imported to the SIGVerse system owing to the format of the mechanism description. Currently, we need to modify the URDF format for manual use in SIGVerse.

The design of software modules to control virtual robots is another limitation. The controller modules in robot simulators, such as Gazebo, are often provided by the manufacturer of the robot and are executed as a process on Ubuntu. However, we have to port the robot controller into C#, which should be executed on Unity. The cost of porting should be discussed when general users employ the SIGVerse system. Four types of robots, HSR (Yamamoto et al., 2018), Turtlebot2, Turtlebot3, PR2 (Wyrobek et al., 2008), and TIAGo (Pages et al., 2016), are currently provided by the developer team.

Another advantage of the proposed SIGVerse system is that the participants can easily participate in experiments in a VR environment. However, an autonomous agent module that acts in a VR environment without real participants/users is not realized. A future research direction is to construct an autonomous agent module based on the analysis of an HRI dataset. The original dataset captured in the HRI experiments and augmented datasets, which could be generated in the VR environment, should be employed in the construction process based on machine learning techniques (Gotsu and Inamura, 2019).

The disparity between the real world and the virtual environment often becomes a discussion focus. Robot motion controlled in a virtual simulator is a reoccurring critical argument in robotics research. Furthermore, the cognitive behavior of the participants is the basis for another discourse. We investigated the difference in human behavior derived from the condition of the HMD’s field of view (FOV) (Mizuchi and Inamura, 2018). Distance perception in the VR environment (Phillips et al., 2010) is another challenge faced when evaluating the HRI in VR. Hence, the appropriate design of the VR environment should be considered, in which participants can behave in a similar way as they would in the real world.

### 5.3 Future Direction of the VR Platform

The current SIGVerse platform operates only in a VR environment; however, *via* the AR function, the applications of virtual robot agents could expand to situations in which they interact with humans in the real world. The human navigation task described in Section 4.1.2 is an example where the AR system can be applied to improve the intelligence of service robots in the real world. This function will be addressed as a future task, as an extensive range of applications can be expected by adjusting the boundary between the virtual environment and the real-world environment according to various situations and tasks.

In the image processing fields, such as in MNIST and ImageNet, many datasets exist for object recognition using machine learning, and a platform that can objectively evaluate the performance of the algorithm proposed by each researcher is provided. Several datasets related to human activity can be adopted. Video clips (Patron-Perez et al., 2012) and motion capture data (Xia et al., 2017) for human movements, natural language sentences for describing movements (Plappert et al., 2016), and conversational data for guiding the user to the destination (Vries et al., 2018) can be employed. However, no dataset in the HRI field contains conversations to manipulate and navigate the object in a complex daily life environment. These datasets are indispensable for promoting the research on interactive intelligent robots in the future, and the VR platform described in this paper is a potential foundation.

## 6 Conclusion

We developed an open software platform to accelerate HRI research based on the integration of the ROS and Unity framework with cloud computing. One of the contributions of this study is the design of SIGVerse as the cloud-based VR platform with high reusability, addressing four significant challenges: cost reduction of experimental environment construction, provision of the same experimental conditions to participants, reproduction of past experiences, and development of a base system for the natural interface of robot/avatar teleoperations. We also demonstrated the feasibility of the platform in three case studies: robot competition, evaluation of the subjective quality of HRI, and motion learning by demonstration, which is the second contribution. In particular, the establishment of a foundation for an objective and fair evaluation of HRI, primarily *via* deployment in robot competitions, demonstrates the future direction and potential for the advancement of the HRI research community. This is the third contribution of this study.

Future intelligent robots will be required to exhibit deep social behaviors in a complex real world. Accordingly, a dataset for learning social behavior and evaluating performance should be established. A simulation environment that allows autonomous robots and real humans to interact with each other in real time is essential for both the preparation of these datasets and the establishment of an objective HRI evaluation. The proposed system, which combines a VR system and a robot simulation *via* cloud computing, is a significant approach to accelerating HRI research.

## Data Availability

The raw data supporting the conclusions of this article will be made available by the authors, without undue reservation. The source code of the proposed system is available on GitHub https://github.com/SIGVerse.

## Footnotes

1
https://github.com/siemens/ros-sharp.

2
https://assetstore.unity.com/packages/tools/newtonvr-75712.

3 World Robot Summit, Service Category, Partner Robot Challenge http://worldrobotsummit.org/en/wrc2018/service/.

4
http://worldrobotsummit.org/download/rulebook-en/rulebook-simulation_league_partner_robot_challnege.pdf.

5
https://sites.google.com/view/robocup-athome-sim/home.

6
https://aws.amazon.com/ec2/.

7
https://aws.amazon.com/.

8
https://www.photonengine.com/en-US/Photon.

9
https://www.photonengine.com/PUN.

10
https://doc.photonengine.com/en-us/realtime/current/getting-started/onpremises-or-saas.
